# A novel low-tech lined bed cultivation enhances drought stress tolerance of cucumber in semi-arid conditions

**DOI:** 10.1038/s41598-026-37245-z

**Published:** 2026-02-12

**Authors:** Ibrahim A. Abouelsaad

**Affiliations:** 1 Horticulture Department, Faculty of Agriculture, Damanhour University, Damanhour, 22516 Egypt; 2 Faculty of Desert Agriculture, King Salman International University, Ras Sedr, 46618 Egypt

**Keywords:** Water retention, Drought stress, Cucumber, Physiological stress, Water use efficiency, Ecology, Ecology, Environmental sciences, Plant sciences

## Abstract

Water scarcity and nutrient leaching limit cucumber (*Cucumis sativus* L.) production in semi-arid sandy soils. This study evaluated a low-tech lined trench-bed system, incorporating polyethylene liners to reduce deep percolation and nutrient loss, under protected cultivation in Nubaria, Egypt (summer season). The study compared two cultivation systems (lined vs. non-lined beds) under two irrigation regimes (100% and 50% ETc; crop evapotranspiration). Lined beds significantly enhanced shoot and root biomass, vine height, leaf number, and leaf area under deficit irrigation, with values comparable to full irrigation, while non-lined beds showed sharp reductions. Foliar N, P, K, Ca, and Mg in lined beds at 50% ETc were statistically similar to 100% ETc, contrasting with 35–55% declines in non-lined beds. Physiologically, lined beds maintained chlorophyll content and leaf relative water content, while minimizing proline and malondialdehyde accumulation, indicating improved osmotic adjustment and membrane stability. Yield was sustained in lined beds under deficit irrigation (26.56 kg m⁻²; 14.4% reduction from 100% ETc) compared to sharp declines in non-lined beds (11.31 kg m⁻²; 63.6% reduction). The highest water use efficiency WUE (98.37 kg m⁻³) occurred in lined beds at 50% ETc, over double the equivalent non-lined treatment. The lined trench-bed effectively preserves growth, nutrient status, physiological integrity, and yield under drought, offering a scalable solution for water-limited horticulture.

## Introduction

Cucumber (*Cucumis sativus* L.) is an economically important vegetable crop that is widely cultivated in semi-arid regions, including under protected greenhouse conditions. However, cucumber production in these water-limited environments faces chronic challenges due to scarce water supplies, poor soil structure, and rapid nutrient leaching^1^. Intensified irrigation demands are depleting local water resources (e.g. aquifers in semi-arid farmlands), making conventional irrigated agriculture unsustainable and driving the need for water-conserving strategies^2^. The shallow root system of cucumber makes this crop particularly vulnerable to drought stress and nutrient loss from the root zone^3^. Consequently, prolonged water deficits can cause significant yield reductions in cucumber. At the same time, excessive irrigation on the coarse-textured soils common in semi-arid areas often leads to fast drainage and leaching of nutrients beyond the root zone, wasting fertilizers and polluting groundwater^4^. These twin problems of limited water and poor soil water-holding capacity underscore the importance of improving water-use efficiency and developing sustainable cultivation systems for cucumber in semi-arid environments.

In arid regions, water-saving techniques have been investigated to boost water use efficiency (WUE) and sustain yields in crops cultivation^5,6^. Mulching is among the most effective methods for improving water management in both open-field and protected cropping^1,7^. Among various mulching techniques, plastic film mulches have demonstrated pronounced agronomic benefits, particularly in enhancing soil moisture retention, moderating soil temperature, suppressing weed proliferation, and consequently improving crop yield and water-use efficiency under semi-arid cultivation conditions^7^. For cucumber, several studies showed that mulching significantly enhanced cucumber yield, leaf nutrient content, and plant growth parameters under water stress, thereby improving WUE compared to bare soil^8^.

Despite the benefits of mulching, this technique has limitations under certain conditions. While plastic and organic mulches effectively reduce surface evaporation and moderate soil temperature, they offer limited control over deep percolation losses, particularly in sandy soils with low water-holding capacity^5,6^. In such soil, a significant portion of irrigation water can infiltrate rapidly beyond the effective rooting depth, rendering surface mulching insufficient to conserve water within the root zone^6^. Additionally, mulches do not prevent nutrient leaching, as soluble fertilizers can still move below the root zone with excess water, leading to reduced nutrient-use efficiency and potential groundwater contamination^4^. Furthermore, improper application of mulches may create soil aeration problems, increase pest and disease incidences beneath the mulch layer, and in some cases, lead to localized overheating of the root zone during high-temperature periods^1,5,9^. On the other hand, researchers have explored soil amendments (e.g. biochar and hydrogels) to improve soil structure and water-holding capacity^10,11^. While amendments like biochar have shown promise in increasing soil moisture retention and thereby aiding crops in water-scarce regions, their effectiveness can vary with soil type, and they often require substantial input rates and long-term investment^10^.

In this context, the current study introduces a low-tech lined trench-bed system as an innovative approach to improve cucumber production under semi-arid conditions. The lined bed system is a straightforward engineering intervention: it involves lining the soil bed or planting trench with an impermeable material to create a partial barrier beneath and around the root zone. Lined beds are fully lined trenches or planting beds, where impermeable liners (e.g., polyethylene sheets) are installed along the trench walls and bottom, creating a complete basin for water retention. The concept is analogous to recently developed subsurface water retention technologies (SWRT), which use U-shaped impermeable membranes buried under crop root zones at variable depths (30–60 cm), arranged in a grid or stripe pattern across the field to hold water and nutrients^12^. Studies on SWRT in other crops have demonstrated enhanced plant performance and drought tolerance by reducing deep percolation, thereby keeping the soil beneath the crop moister and more nutritionally enriched^12,13^. The lined bed system seeks to achieve similar benefits, increasing the effective water supply in the root environment and minimizing nutrient leaching, but with a low-cost, easily deployable design suited for smallholder or low-tech settings. By maintaining a moist, nutrient-rich root zone, the lined bed is expected to alleviate water stress on the crop and improve fertilizer use efficiency without the need for sophisticated irrigation infrastructure. The application of a lined trench-bed for horticultural crop production has yet to be explored in scientific literature, establishing this study as a pioneering effort toward advancing sustainable and resilient cultivation systems in water-scarce environments.

The present study was undertaken to evaluate the efficacy of the low-tech lined bed system in enhancing cucumber growth and yield under semi-arid conditions. The current study aimed to quantify the impacts of the lined bed on cucumber performance, including vegetative growth, physiological responses, nutrient uptake, and fruit yield as well as on irrigation water productivity, in comparison to conventional (unlined) beds. The trials were conducted under varying irrigation levels to assess whether the lined bed can mitigate water stress and maintain productivity even when water supply is limited. The study hypothesized that the lined bed system, by improving root-zone water retention, would support better cucumber growth and higher water-use efficiency under deficit irrigation, relative to standard cultivation. This introduction of a simple yet innovative lined trench-bed approach is envisioned as a contribution to ongoing efforts to improve crop water productivity and sustainability in protected agriculture under water-limited conditions. The findings from this study will help determine if such a low-cost system can significantly boost cucumber production and resource-use efficiency, thereby offering a new tool for growers in semi-arid regions striving to conserve water while sustaining high yields.

## Materials and methods

### Plant materials and experimental conditions

The present study was conducted during the summer season from May to August 2024 at Nubaria region, Beheira Governorate, Egypt (30.3400° N, 30.2900° E). The experiment aimed to compare the effects of an underground trench system with lining against a conventional open-bed soil cultivation method on cucumber growth, water management, and physiological performance under drought stress within protected cultivation. Baseline soil characterization was conducted prior to planting. Composite samples from the 20 cm layer (root zone) were collected and analyzed for texture, pH, electrical conductivity, and macronutrient concentrations following standard protocols^14,15^. The site soil was identified as sandy, and slightly alkaline, with low organic matter and poor water-holding capacity, typical of reclaimed desert soils. Detailed physical and chemical properties of soil are presented in Table 1.

**Table 1:** Physical and chemical properties of the experimental soil.

Property	Unit	Value
Physical properties		
Sand	%	94.50
Silt	%	3.71
Clay	%	1.79
Soil texture	–	Sand
Bulk density	g cm⁻³	1.61
Field capacity	% (v/v)	8.11
Chemical properties		
pH	–	7.81
Electrical conductivity (EC)	dS m⁻¹	2.11
Organic matter	%	0.39
Calcium carbonate (CaCO₃)	%	2.20
Calcium (Ca²⁺)	meq L⁻¹	9.20
Magnesium (Mg²⁺)	meq L⁻¹	9.10
Potassium (K⁺)	meq L⁻¹	1.50
Sodium (Na⁺)	meq L⁻¹	13.70
Sulfate (SO₄²⁻)	meq L⁻¹	22.10
Chloride (Cl⁻)	meq L⁻¹	5.90

Seeds of *Cucumis sativus* L., cv. Dacnis F1 (Syngenta Vegetable seeds Company, Egypt), were germinated in foam trays filled with a 1:1 (v/v) peat moss and vermiculite mixture. During the nursery stage, seedlings were fertigated using a complete soluble fertilizer containing macro- and micronutrients at a concentration of 2 g L⁻¹. After 20 days, when seedlings reached the 2 to 3 true-leaf stage, uniform plants were transplanted into the experimental plots. The experiment was carried out inside a naturally ventilated shading net house with a total area of 135 m². The structure was dome-shaped, measuring 9 m in width, 15 m in length, with sidewalls of 2.5 m and a central ridge height of 3.5 m. It was constructed from galvanized iron pipes (2 mm thickness) and covered with a white polyethylene shade net providing 50% light interception. No active environmental controls were used; temperature and humidity were moderated solely through passive ventilation and shading. The experimental site is characterized by a hot semi-arid climate with high evaporative demand, particularly during the summer months^16^, which makes it an ideal location for evaluating water-efficient cultivation systems under drought conditions. The microclimatic data (monthly average air temperature and relative humidity) inside the shading net house during crop growth are presented in Table 2.

**Table 2:** Monthly average air temperature and relative humidity inside the protected structure during the growing season.

Month	Average temperature (°C)	Average relative humidity (%)
May	26.1	47.6
June	29.0	48.9
July	30.1	51.4
August	30.7	53.7

### Treatments and experimental design

**Fig. 1:**
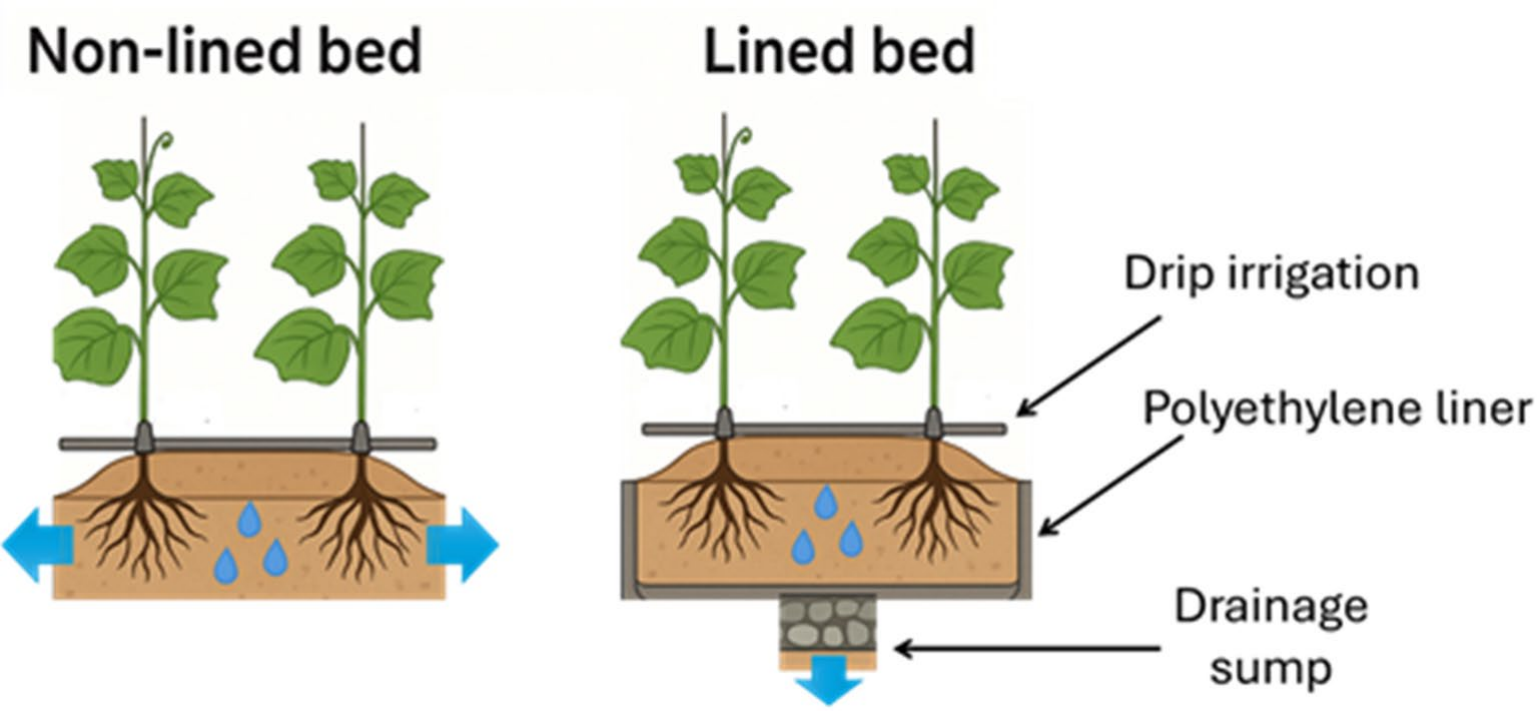
Comparative schematic illustration of non-lined and lined raised-bed systems used for cucumber cultivation in reclaimed sandy soil under protected conditions.

**Fig. 2:**
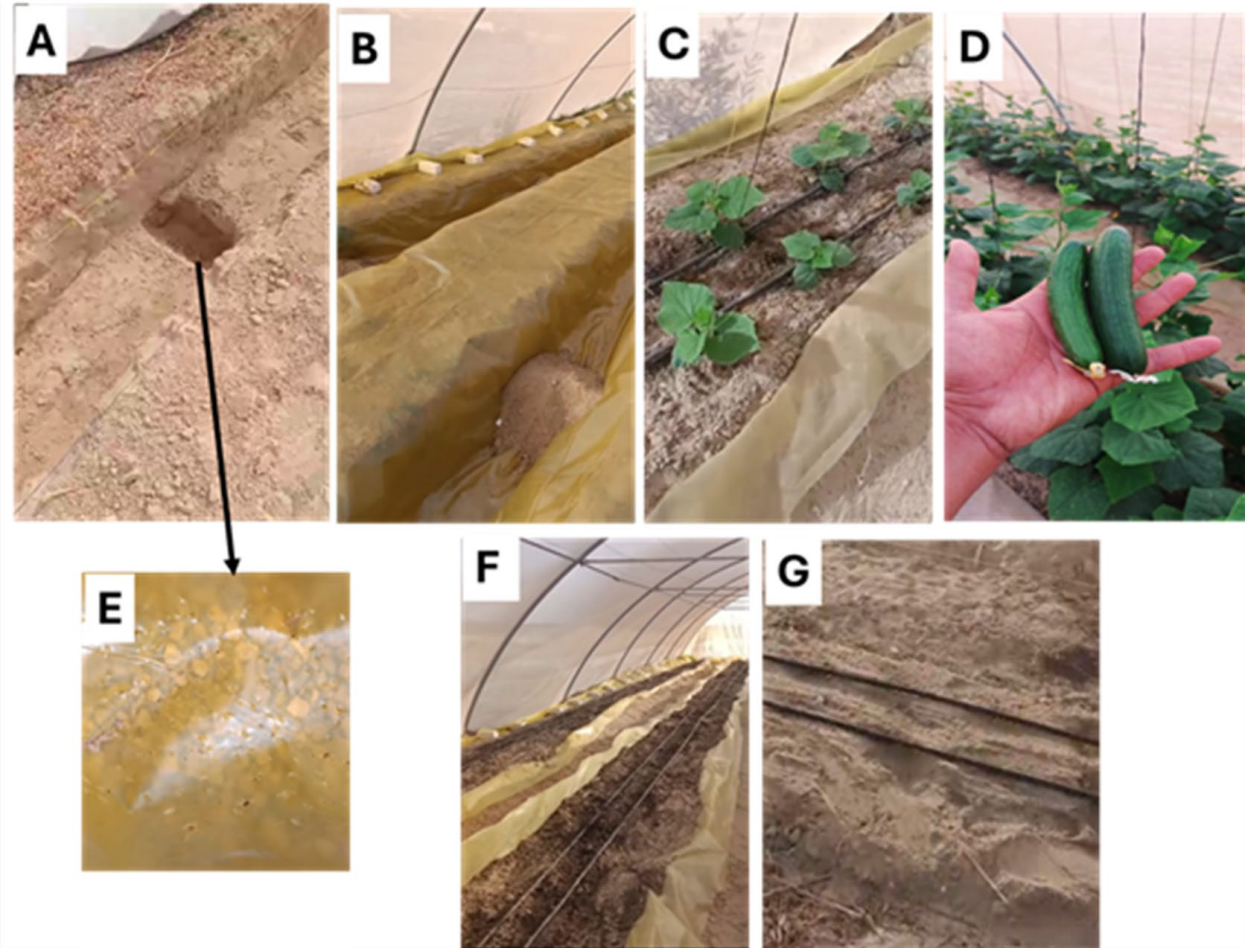
Sequential steps and infrastructure of the lined trench-bed system used in cucumber cultivation under protected environment conditions in sandy soil. (**A**) Excavation of the trench (0.6 m W× 0.5 m D) in sandy soil. (**B**) Installation of polyethylene liner (200 μm) in the lined bed. (**C**) Early vegetative stage of cucumber seedlings (cv. Dacnis F1) transplanted on lined beds. (**D**) Fruit harvest form lined beds showing marketable cucumber yield. (**E**) Close-up view showing coarse gravel within the sump for lined bed drainage. (**F**) Prepared lined bed prior to transplantation showing drip lines. (**G**) Comparative view of the conventional non-lined (open) bed under the same irrigation setup.

For the underground trench system with impermeable lining (lined bed), trenches were excavated to dimensions of 0.6 m (width) × 0.5 m (depth). Each trench was equipped with a drainage sump (20 cm × 20 cm × 20 cm) positioned at the base at 2-meter intervals, filled with coarse gravel. The drainage sump was installed to prevent water stagnation beneath the polyethylene liner and facilitate excess water removal. A high-density polyethylene liner (200 microns) was installed throughout the trench to minimize seepage and enhance water retention in the root zone. The trench was then backfilled with the original soil to form a raised bed approximately 15 cm above the surrounding surface, aligned in height with the conventional open-bed (non-lined bed) plots (Figs. 1 and 2).

The experimental layout followed a split-plot arrangement with the cultivation system as the main plot factor and irrigation level as the subplot factor. The experiment included four treatments derived from the factorial combination of two cultivation systems (lined beds and non-lined beds), each subjected to two different irrigation regimes. Cucumber plants were subjected to two distinct irrigation regimes corresponding to 50% and 100% of crop evapotranspiration (ETc), which amounted to 271 L/m² and 542 L/m², respectively, over the entire growing season. These irrigation volumes were applied via a drip irrigation system starting from the day of transplanting. The daily irrigation volumes (L/m²/day) were calculated based on ETc, adjusted using crop coefficient (Kc) values specific to each growth stage^17^.The methodology for determining ETc, Kc values, and irrigation volumes followed the procedures outlined by Kumar et al. ^18^. Irrigation volumes for each cultivation system were precisely quantified using calibrated volumetric flow meters, ensuring accurate measurement of total water applied throughout the experimental period.

Each experimental unit, whether a lined or non-lined bed, consisted of a plot measuring 7.0 m in length and 0.6 m in width. To avoid hydraulic interference, a 1.0 m buffer was maintained between adjacent plots. The study was conducted with three replicates per treatment, resulting in a total of 12 plots. All beds were equipped with drip irrigation lines (16 mm diameter), laid in parallel at 40 cm spacing. Seedlings were planted on both sides of each drip line at 40 cm intervals, resulting in a final spacing of 40 cm × 40 cm and a planting density of 8.10 plants m⁻². Each plot contained 34 plants arranged in two paired rows. All plants were vertically trained using plastic twine attached to overhead trellising wires and maintained as single-leader vines through periodic removal of axillary shoots. A commercial organic fertilizer was incorporated into the soil of each bed at a rate of 2 kg m⁻² prior to transplanting, ensuring uniform distribution and enhancing baseline soil fertility. Throughout the entire growing period, cucumber plants were fertigated using a nutrient solution formulated according to the guidelines in Nutrient Solutions for Greenhouse Crops^19^. A 2,000-liter (2.0 m³) polyethylene tank was used to prepare and supply the fertigation solution, which was delivered to the crop via a drip irrigation system. Electrical conductivity (EC) and pH of the nutrient solution were monitored and maintained within optimal ranges (EC: 1.2–1.4 dS m⁻¹; pH: 5.8–6.2). The chemical composition of the nutrient solution used in the fertigation tank is presented in Table 3.

**Table 3:** Composition of the nutrient solution used for cucumber fertigation during the experimental period^19^.

Element	Concentration (mg/L)	Fertilizer source
Nitrogen (N)	180	Calcium nitrate and Potassium nitrate
Phosphorus (P)	50	Monopotassium phosphate
Potassium (K)	200	Potassium nitrate
Calcium (Ca)	150	Calcium nitrate
Magnesium (Mg)	50	Magnesium sulfate
Sulfur (S)	60	Potassium sulfate
Iron (Fe)	2.5	Fe-DTPA chelate
Manganese (Mn)	0.5	Mn-EDTA chelate
Zinc (Zn)	0.1	Zn-EDTA chelate
Copper (Cu)	0.05	Cu-EDTA chelate
Boron (B)	0.5	Boric acid
Molybdenum (Mo)	0.05	Sodium molybdate

### Growth trait assessment

Growth-related parameters were measured at 70 days after transplanting. A total of six representative plants per treatment (two plants per replicate) were randomly selected for each plot. Plants were carefully uprooted to preserve root integrity and gently washed to remove adhering soil particles. Shoot and root tissues were separated, and fresh samples were oven-dried at 70 °C to constant weight (72 h) using a forced-air convection oven to determine shoot dry weight and root dry weight (g plant⁻¹). Plant height (cm plant⁻¹) was measured from the soil surface to the apical meristem using a graduated measuring pole. The number of fully expanded leaves per plant was counted manually. Leaf area per plant was estimated using a non-destructive length–width model validated for cucumber leaves, using the formula:

$${\text{Leaf Area}}={\mathrm{L}} \times {\mathrm{W}} \times 0.{\mathrm{75}}$$

where *L* is leaf length, *W* is maximum width, and 0.75 is the species-specific correction factor^20^. Root length (cm plant⁻¹) was determined by measuring the longest intact primary root using a flexible ruler.

### Leaf nutrients content

Leaf samples were collected from fully expanded leaves, 70 days after transplanting. For each plot, composite leaf samples (*n* = 5 plants per replicate) were harvested, washed thoroughly with deionized water, and oven-dried at 70 °C to constant weight. The dried samples were ground to a fine powder and analyzed for macronutrient contents. Total nitrogen (N) was determined using the semi-micro Kjeldahl method^21^, while phosphorus (P) was assessed by the molybdenum blue spectrophotometric protocol^22^. Potassium (K), calcium (Ca), and magnesium (Mg) were measured using flame photometry and atomic absorption spectrophotometry (AAS), respectively^14,23^ .

### Measurement of stress physiological parameters

At 70 days after transplanting, relative chlorophyll content (SPAD) was measured in situ using a portable SPAD-502 Plus chlorophyll meter (Konica Minolta, Japan). Three readings per leaf were averaged to obtain a single SPAD value per plant. Leaf relative water content (LRWC) was determined using the standard gravimetric method. Leaf discs were collected and immediately weighed to obtain fresh weight (FW), then floated in distilled water for 4 h under ambient light to determine turgid weight (TW), and subsequently oven-dried at 70 °C for 48 h to record dry weight (DW). LRWC was calculated using the formula: LRWC (%) = [(FW − DW)/(TW − DW)] × 100^24^ .

Free proline content, approximately 0.5 g of fresh leaf tissue was homogenized in 3% sulfosalicylic acid, and the extract was reacted with acid-ninhydrin reagent. Absorbance was read at 520 nm using a UV–Vis spectrophotometer. Results were expressed as µmol g⁻¹ FW using a standard curve prepared with L-proline^25^. Malondialdehyde (MDA) content was estimated as a marker of lipid peroxidation, according to the thiobarbituric acid (TBA) method^26^. Briefly, 0.5 g of fresh leaf tissue was extracted with 0.1% trichloroacetic acid (TCA), followed by reaction with 0.5% TBA in 20% TCA and incubation at 95 °C for 30 min. Absorbance was measured at 532 nm and corrected for nonspecific turbidity at 600 nm. MDA concentration was expressed as µmol g⁻¹ FW using an extinction coefficient of 155 mM⁻¹ cm⁻¹.

### Yield and water use efficiency (WUE)

Each plot consisted of a defined area (4.2 m²) planted with a standard commercial cucumber cultivar at optimal density. Fruits were harvested at commercial maturity, and fruit number per plant and total yield (kg m⁻²) were recorded over the production cycle (85 days after transplanting). Yield reduction (%) was calculated relative to the highest yielding treatment (lined bed with 100% ETc). The total water applied (m³ m⁻²) was recorded for each treatment using calibrated flow meters installed on the drip lines. Water use efficiency (WUE) was computed as the ratio of total marketable yield to total applied irrigation water, expressed in kg m⁻³^27^.

### Statistical analysis

A two-way analysis of variance (ANOVA) was performed to evaluate the main and interactive effects of the cultivation system and irrigation level on all measured traits. The experimental design was a split-plot arrangement with cultivation system as the main plot factor and irrigation level as the subplot factor, replicated three times. Treatment means were compared using Tukey’s Honest Significant Difference (HSD) test at a significance threshold of *P* ≤ 0.05 to detect statistically significant differences between treatments. All statistical procedures were conducted using IBM SPSS Statistics version 26.0 (IBM Corp., Armonk, NY, USA).

## Results

### Vegetative growth parameters

Both the cultivation system (CS) and irrigation level (IL) had highly significant effects on cucumber vegetative growth, with significant interactions between factors for all parameters except root length (Table 4). Plants grown on lined beds showed greater shoot and root biomass, taller vines, more leaves, and larger leaf area compared to those on non-lined beds (CS main effect, *P* ≤ 0.001 for all these traits). Likewise, well-watered plants (100% ETc) exceeded deficit-irrigated plants (50% ETc) in all growth attributes (*P* ≤ 0.001). However, the lined bed system largely mitigated growth reductions under limited irrigation (significant CS × IL interaction, *P* ≤ 0.001). In lined beds, there were no significant differences between 50% and 100% ETc for shoot dry weight, plant height, leaf number, or leaf area, whereas in non-lined beds the 50% ETc treatment caused marked declines in these parameters (Table 4). For example, shoot dry weight, vine length, and leaf area in non-lined beds under 50% ETc were approximately 30–50% lower than their 100% ETc counterparts, while in lined beds these metrics under deficit irrigation remained statistically similar to the fully irrigated control (Table 4). Root length exhibited a distinct response: it was not significantly affected by cultivation system (CS effect not significant) or the CS × IL interaction but was significantly influenced by irrigation level alone. Across both bed systems, deficit irrigation induced longer roots (*P* ≤ 0.01), with roots under 50% ETc extending approximately 15% longer than those under full irrigation (Table 4).

**Table 4:** Effects of cultivation system and irrigation level on growth parameters of cucumber plants.

Cultivation system (CS)	Irrigation level (IL)	Shoot dry weight (g plant^− 1^)	Root dry weight (g plant^− 1^)	Plant height (cm plant^− 1^)	No. of leaves (plant^− 1^ )	leaves area (dm^2^ plant^− 1^)	Root length (cm plant^− 1^)
Lined bed	100% ETc	67.01 ± 3.18^a^	6.38 ± 0.47^a^	186.5 ± 6.56^a^	34.7 ± 3.06^a^	49.56 ± 3.64^a^	32.50 ± 2.00^a^
	50% ETc	64.94 ± 1.08^a^	6.47 ± 0.14^a^	180.6 ± 4.37^a^	34.3 ± 2.08^a^	45.64 ± 2.70^a^	37.10 ± 1.44^b^
Non-lined bed	100% ETc	68.02 ± 1.67^a^	6.15 ± 0.44^a^	187.1 ± 7.32^a^	36.0 ± 2.00^a^	46.84 ± 2.00^a^	32.50 ± 1.32^a^
	50% ETc	35.57 ± 2.36^b^	4.48 ± 0.47^b^	133.8 ± 7.18^b^	25.3 ± 2.52^b^	30.11 ± 1.84^b^	38.17 ± 1.04^b^
Significance level							
CS		***	***	***	***	***	NS
IL		***	***	***	***	***	**
CS × IL		***	***	***	***	***	NS

Values are presented as means ± standard error (SE). Means within the same column followed by different letters are significantly different at *P* ≤ 0.05 according to Tukey’s HSD test. Significance levels are indicated as follows: **Highly significant at *p* ≤ 0.01; ***Very highly significant at *p* ≤ 0.001; NS = not significant.

### Leaf nutrient content

**Fig. 3:**
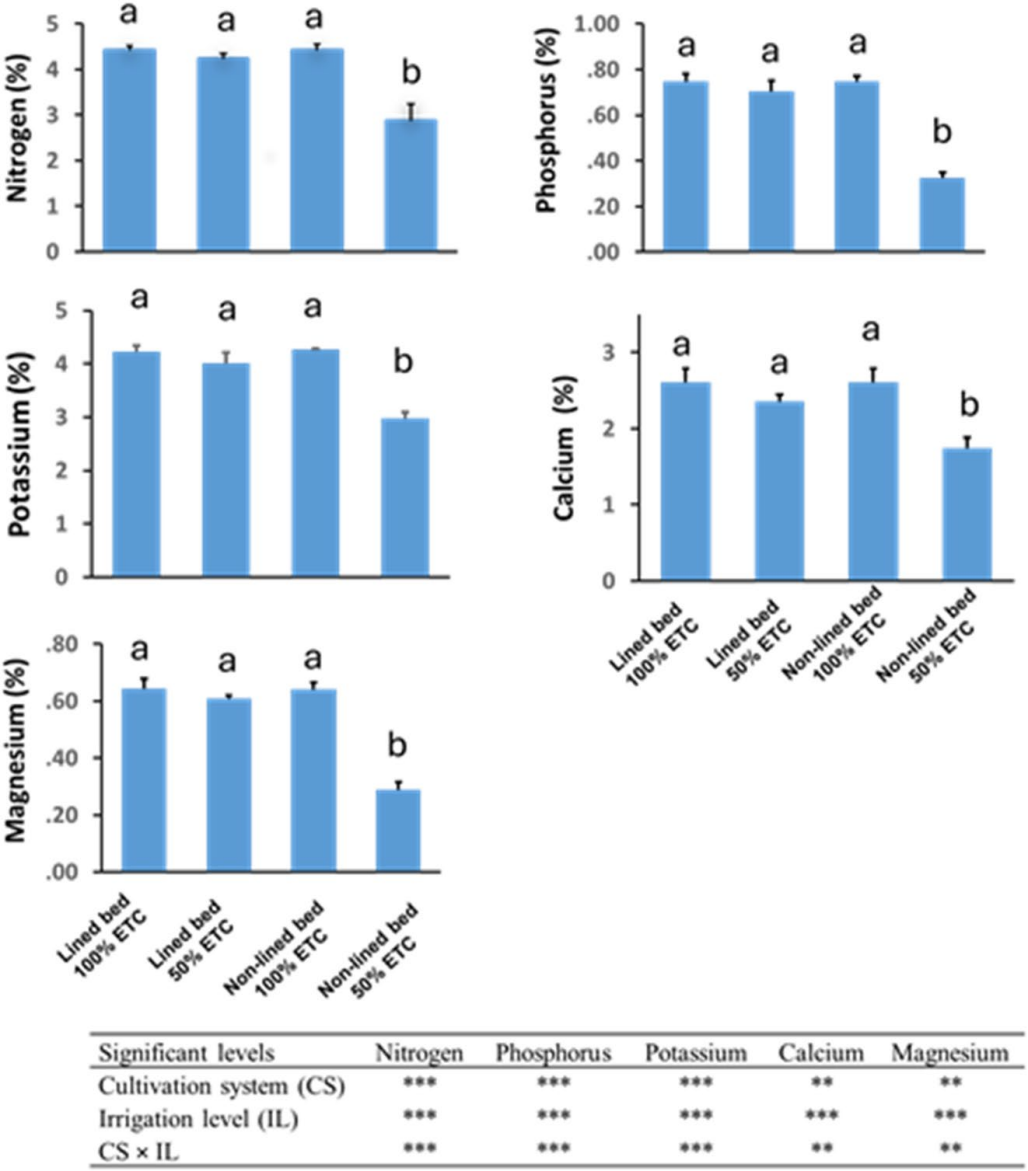
Effect of cultivation system and irrigation level on nutrient contents in cucumber leaves. Bars represent means ± SE. Different letters indicate significant differences at *P* ≤ 0.05 according to Tukey’s HSD test. Analysis of variance (ANOVA) showing the significance levels of the main effects and their interaction on nutrient contents. Significance levels are indicated as follows: **Highly significant at *p* ≤ 0.01; ***Very highly significant at *p* ≤ 0.001.

All measured macronutrients (N, P, K, Ca, and Mg) showed highly significant main effects of cultivation system and irrigation level, as well as CS × IL interactions (Fig. 3). In general, plants grown on lined beds had higher foliar N, P, K, Ca, and Mg contents than those on non-lined beds, and well-watered plants (100% ETc) had higher nutrient levels than plants under deficit irrigation (50% ETc).

Under severe deficit (50% ETc) in the non-lined beds, leaf nutrient levels dropped sharply. For example, in non-lined 50% ETc plants, leaf N fell to 2.88% (compared to 4.41% under 100% ETc), P to 0.33% (vs. 0.75%), and K to 2.97% (vs. 4.26% in the non-lined 100% control) (Fig. 3). These values represent reductions of approximately 35–55% in N, P, and K relative to well-watered conditions. Foliar Ca and Mg were likewise lowest in non-lined plants under 50% ETc (Ca 1.75%, Mg 0.29%), about one-third lower than the corresponding full irrigation values (Fig. 3). In contrast, cucumber plants grown on lined beds maintained much higher nutrient status under water stress. Leaves of lined-bed plants at 50% ETc still contained 4.24% N, 0.70% P, 4.02% K, 2.35% Ca, and 0.61% Mg – concentrations only slightly reduced from the 100% ETc lined treatment, and not significantly different from the fully irrigated plants (Fig. 3). Consequently, the lowest leaf nutrient contents were recorded in the non-lined 50% ETc treatment for all nutrients, whereas the highest values were generally observed in well-watered plants (both bed types being similar when irrigation was ample).

### Stress physiological parameters

**Fig. 4:**
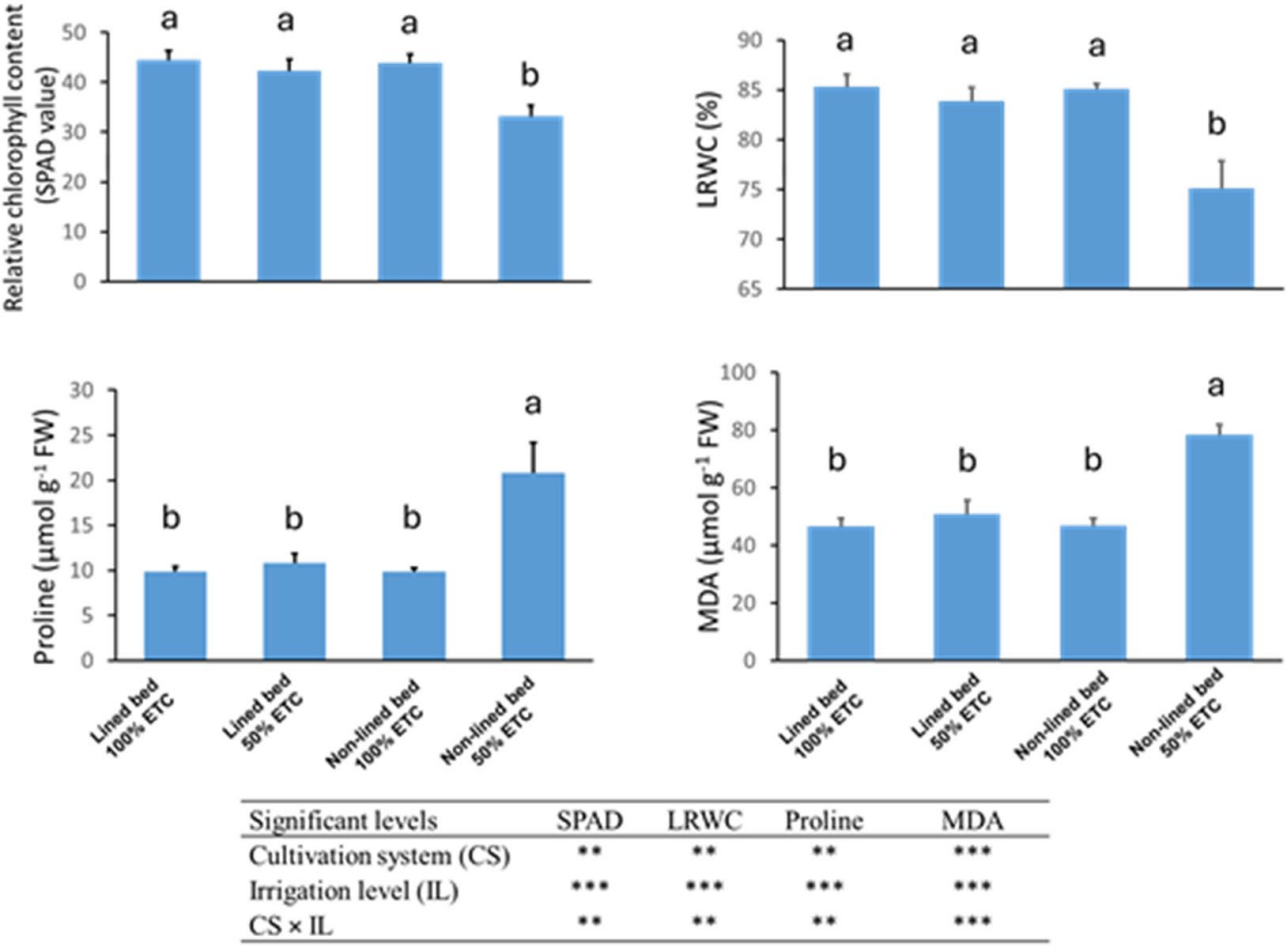
Effect of cultivation system and irrigation level on relative chlorophyll content (SPAD), leaves relative water content (LRWC), proline, malondialdehyde (MDA) in cucumber leaves. Bars represent means ± SE. Different letters indicate significant differences at *P* ≤ 0.05 according to Tukey’s HSD test. Analysis of variance (ANOVA) showing the significance levels of the main effects and their interaction on physiological traits. Significance levels are indicated as follows: **Highly significant at *p* ≤ 0.01; ***very highly significant at *p* ≤ 0.001.

Significant main effects of cultivation system and irrigation level were observed for all measured physiological traits, and each trait showed a highly significant CS × IL interaction (Fig. 4). Plants on lined beds had higher relative chlorophyll content (SPAD values) and leaf relative water content (LRWC) than those on non-lined beds, and well-watered plants had higher SPAD and LRWC than deficit-irrigated plants (Fig. 4). In lined beds, SPAD (42.30) and LRWC (83.87%) under 50% ETc remained comparable to the 100% ETc treatment (44.40 and 85.30%, respectively), with no significant difference between irrigation levels. In contrast, non-lined bed plants at 50% ETc exhibited much lower SPAD (33.23) and LRWC (75.01%), a reduction of about 24% and 12%, respectively, relative to the well-watered control (Fig. 4). Thus, the lowest chlorophyll content and water status occurred in the non-lined 50% ETc treatment, whereas lined bed plants largely maintained their leaf greenness and hydration even under reduced irrigation.

Proline and malondialdehyde (MDA) showed the opposite trend, accumulating significantly under water deficit especially in the conventional beds. Irrigation at 50% ETc led to elevated proline and MDA levels in leaves (IL main effect *P* ≤ 0.001), and the increase was exacerbated in the non-lined system (CS × IL interaction; Fig. 4). In non-lined beds, water-stressed plants accumulated approximately double the proline and MDA compared to well-watered plants. Leaf proline in the non-lined 50% ETc treatment reached 20.83 µmol g^−1^ FW, and MDA rose to approximately 78 µmol g^−1^ FW, versus 9.88 µmol g^−1^ FW proline and 46.77 µmol g^−1^ FW MDA under 100% ETc (Fig. 4). By contrast, lined bed plants under 50% ETc had only slight, non-significant increases in these stress indicators: their proline (10.79 µmol g^−1^ FW) and MDA (50.75 µmol g^−1^ FW) remained low and statistically similar to the fully irrigated treatments.

### Yield performance and water use efficiency

The number of fruits per plant, total yield per m^2^, and irrigation water use efficiency (WUE) all showed significant effects of cultivation system (*P* ≤ 0.01) and irrigation level (*P* ≤ 0.001), with highly significant CS × IL interactions for each trait (*P* ≤ 0.001; Table 5). Under full irrigation (100% ETc), the lined and non-lined bed treatments produced statistically equivalent yields and fruit counts. Mean total yield was 31.04 kg m^−2^ in the lined bed vs. 30.27 kg m^−2^ in the non-lined bed, with each producing approximately 40 marketable fruits per plant (Table 5).

**Table 5:** The impact of cultivation system and irrigation level on fruit number, total yield, reduction in yield, total applied water, and water use efficiency.

Cultivation system (CS)	Irrigation level (IL)	Fruit number (plant^− 1^)	Total yield (kg m^− 2^)	Reduction in yield (%)	Total water applied (m^3^ m^− 2^)	Water use efficiency (Kg m^− 3^)
Lined bed	100% ETc	40.0 ± 4.58^a^	31.04 ± 1.02^a^	0.00	0.541	57.30 ± 1.62^b^
	50% ETc	35.0 ± 1.73^a^	26.56 ± 0.76^b^	14.43	0.270	98.37 ± 2.42^a^
Non-lined bed	100% ETc	39.7 ± 3.51^a^	30.27 ± 1.12^a^	2.48	0.541	55.95 ± 1.78^b^
	50% ETc	17.3 ± 1.52^b^	11.31 ± 1.51^c^	63.56	0.270	41.88 ± 4.80^c^
Significance level						
CS		**	**			**
IL		***	***			***
CS × IL		***	***			***

Values are presented as means ± standard error (SE). Means within the same column followed by different letters are significantly different at *P* ≤ 0.05 according to Tukey’s HSD test. Significance levels are indicated as follows: **highly significant at *p* ≤ 0.01; ***very highly significant at *p* ≤ 0.001.

When irrigation was reduced to 50% ETc, however, the yield in the conventional non-lined beds dropped dramatically, whereas the lined beds sustained a high yield. Cucumber plants in lined beds at 50% ETc produced 26.56 kg m^− 2^ of fruit, only a 14.4% yield reduction relative to the 100% ETc lined treatment (31.04 kg m^− 2^; Table 5). Fruit number in lined 50% ETc was about 35 per plant, not significantly different from the well-watered crop (40 fruits). In sharp contrast, the non-lined bed with 50% ETc irrigation yielded just 11.31 kg m^− 2^. This was a 63.56% decrease compared to the lined bed 100% ETc yield and corresponded to only 17.3 fruits per plant under deficit, less than half the fruit count of the well-watered control. The highest WUE was achieved in the lined bed at 50% ETc, at 98.37 kg of yield per m^3^ water, which was more than double the WUE of the non-lined 50% ETc treatment (41.88 kg m^− 3^) and also significantly higher than the WUE of 100% ETc treatment (Table 5).

## Discussion

The novel lined trench-bed system markedly influenced cucumber vegetative growth in the semi-arid, shade-net house environment. In general, plants grown in lined beds exhibited greater shoot and root biomass, taller vines, and more abundant foliage (leaf number and area) compared to those in unlined beds under water-deficit conditions (50% ETc). These results support the hypothesis that lining the root-zone can mitigate drought stress by retaining moisture and nutrients, thereby sustaining vegetative growth even when irrigation is reduced. By reducing deep percolation, the liners likely maintained a more favorable soil moisture around roots, enabling lined-bed plants to maintain turgor and growth processes^12^. This interpretation aligns with root-zone restriction or water-harvesting concepts reported in other contexts. For example, the reduced irrigation in open-field cucumbers, vegetative growth parameters declined significantly, but conserving soil moisture (with mulch) lessened these impacts^7^. Also, grafted cucumber experiments enhancing the root zone environment (via vigorous rootstocks) maintained higher leaf area, leaf count, and plant height under drought^27,28^. In the current study, water availability strongly affected the plants’ nutrient status (leaf N, P, K, Ca, Mg content), and the lined beds helped preserve nutrient uptake under deficit irrigation (50% ETc). This suggests that lining curtails nutrient leaching and enhanced nutrient retention in the root zone. Prior research reported that severe water stress can impair nutrient uptake in plants^29^. The reduction in irrigation (25%) significantly decreased leaf concentrations of N, P, K, Ca, and Mg in field-grown cucumber^7^. This was attributed to reduced mass flow of nutrients to roots and possible inhibition of root activity under drought^30^. In the current experiment, however, the lined treatment likely maintained higher soil moisture and thus nutrient mobility around roots, preventing such drastic nutrient depletion. By minimizing leaching and ensuring closer root contact with nutrients, the lined trenches helped cucumbers sustain a better nutritional status under deficit irrigation than would be expected in a conventional bed. Similar outcomes have been observed with other water-saving techniques, such as soil mulching, which can concentrate nutrients within the wetted root zone and enhance nutrient uptake efficiency^1,6^. Altogether, the findings suggest that the lined trench-bed system not only conserves water but also preserves soil fertility for the crop, a critical benefit for sustainable production in nutrient- and water-limited regions.

Physiological stress indicators measured in the cucumbers further illustrate how lined beds improved drought tolerance. Under 50% ETc irrigation, plants in unlined beds experienced classic drought stress symptoms: lower leaf relative water content (LRWC), reduced chlorophyll (SPAD) readings, and elevated stress metabolites (proline) and oxidative damage markers (malondialdehyde, MDA)^29^. Drought typically causes a decline in LRWC as soil moisture drops, leading to stomatal closure and reduced leaf turgor^31^. In contrast, lined plants under 50% ETc avoided the severe loss of LRWC and chlorophyll that often accompanies dehydration. These trends indicate that the lined root-zone helped plants sustain better hydration and cellular function during the water deficit. Similarly, in nongrafted or unprotected cucumber plants, drought stress has been shown to induce chlorophyll degradation, resulting in reduced SPAD values^27^.

Proline is a well-known osmolyte that accumulates under water stress, helping cells retain water and stabilize proteins^29^. In a previous study, reducing irrigation from full to 75% of ETc in cucumber plants approximately doubled the proline content in the leaves^27^. The current experiment showed that unlined 50% ETc plants likely showed a sharp rise in leaf proline, reflecting greater stress. However, the lined-bed cucumbers likely did not need to synthesize as much proline as their unlined counterparts because their water status was better, an indicator of stress alleviation. Also, lined-bed plants under deficit water had lower MDA levels, signifying less oxidative damage to membranes^31^. In contrast, unlined-bed plants under 50% ETc likely experienced oxidative stress from dehydration, as evidenced by higher MDA. This result echoes findings in similar studies where improving soil moisture availability (via grafting and mulching) correlates with lower accumulation and less lipid peroxidation in droughted plants^6,27^. In summary, the lined trench-bed enabled cucumbers to maintain a more favorable physiological status under limited irrigation, preserving leaf turgor and chlorophyll, and limiting stress-induced biochemical disruptions. These physiological indicators reinforce that the lined bed improved the crop’s drought tolerance at the cellular level.

The lined beds substantially improved fruit yields under water limitation and greatly increased irrigation water use efficiency (WUE). In practical terms, lining the beds enabled a 50% reduction in water supply to achieve nearly comparable yields to the 100% ETc unlined control. This finding holds significant implications for agricultural production in water-scarce environments. In a previous study, in a semi-arid climate, cucumber yields showed 14% reduction when water is cut by 20%, and even a 40% irrigation led to 34% yield loss in their trials^2^. In current study, under 50% ETc, the lined treatment produced a greater number of fruits per plant and a higher total fruit weight compared to the unlined treatment, suggesting that the improved performance was partly attributable to enhanced water retention and more efficient water utilization, which compensated for a portion of the reduced irrigation.

The ratio of yield to water consumed provides a key indicator of sustainable agricultural productivity, especially under water-limited conditions^32^. In the lined plots, yield was maintained much better relative to water input, leading to large improvements in WUE. The lined 50% ETc treatment produced a high yield with half the water, meaning growers could harvest more kilograms of cucumber per cubic meter of water used. Another study in hot climate reported that a moderate deficit (80% ETc) inside a well-ventilated polyhouse led to higher yield than even full irrigation in a shade-net house and concurrently raised water productivity by 20–94% compared to less optimized structures^18^. This underlines that maximizing yield per drop often involves a combination of slight stress (to avoid luxury water consumption) and a controlled root-zone environment, exactly what the lined beds provided. It is worth noting that over-irrigation can sometimes reduce yields due to leaching nutrients. Multiple studies demonstrate that applying deficit irrigation at 80% Ec often yields nearly equal crop production to full irrigation, while substantially improving water use efficiency^33^.

For practical decision-making, a simple ballpark estimate of the costs associated with subsurface polyethylene lining can be expressed on an area basis. Based on material and labor requirements reported for low-input horticultural systems, the initial installation cost may be estimated at approximately USD 2.5–4.0 m². This cost comprising polyethylene liner material (USD 0.5 m²) and labor for trench excavation and installation (USD 2.0–3.5 m^2^). Moreover, the liner is not intended for single season use; when properly installed and protected, it can be reused over multiple cropping cycles, thereby distributing the initial investment over time. When compared with typical gains in marketable yield, improved water-use efficiency, and reduced nutrient losses in sandy soil, such costs fall within a range that may be economically justified for high-value vegetable production. Nevertheless, actual feasibility remains site-specific and depends on local material prices, labor rates, crop value, and irrigation water scarcity.

For growers in arid and semi-arid regions, where water is the scarcest input, adopting a lined trench-bed system could allow substantial water savings (in the current study, up to 50% less water) with only minor yield reduction. This improves water irrigation productivity, which is increasingly emphasized as a sustainability metric. The lined bed system is relatively low-tech and could be implemented using locally available liner materials (plastic sheets, geotextiles) placed in planting trenches, combined with drip. It could be especially useful in protected cultivation setups (like shade-net house or polyhouses), where controlling the root environment is feasible on a small scale. An additional advantage of the lined bed system is its potential long-term durability; because the polyethylene sheet is buried beneath the soil surface, it remains shielded from direct UV radiation, substantially reducing photo-degradation and enabling the structure to remain functional for many years without significant loss of performance.

Despite the clear agronomic benefits observed in the present study, the long-term environmental implications of using polyethylene liners beneath the root zone warrant careful consideration. Improper management of buried plastic materials may contribute to soil degradation through fragmentation and accumulation of microplastics, particularly under repeated cultivation cycles^34^. To mitigate these risks, the use of alternative barrier materials, such as biodegradable or bio-based barrier materials also represent promising research direction, although their long-term performance and degradation dynamics under hot, sandy soil conditions require further validation. Also, the potential risk of water stagnation beneath a subsurface liner, which could create a damp microenvironment conducive to fungal diseases, must be carefully considered when applying this technique. In the present study, no symptoms of waterlogging or disease outbreaks were observed throughout the growing period, which can be attributed to the high drainage capacity of the sandy soil and the inclusion of gravel-filled drainage sumps that facilitated excess water removal and maintained adequate root-zone aeration. These findings indicate that the system is particularly suitable for sandy soil with high permeability, provided that proper drainage outlets are installed at the base of the lined trench and irrigation is carefully managed to avoid over-application. Therefore, the use of bottom drainage holes, regular inspection of drainage functionality, cautious irrigation scheduling, and continuous disease monitoring is strongly recommended. It would also be valuable to monitor plant root distribution in lined vs. unlined beds, possibly using root imaging or excavation, to confirm how roots behave when lateral spread is encouraged but downward movement is blocked. In cucumbers, which naturally have relatively shallow roots, the trench depth (50 cm) was likely not restrictive, but this could be studied with plants with larger roots.

One more practical consideration is the potential long-term effect on soil. Since the liner prevents deep percolation, salts from fertilizer or irrigation water could accumulate in the root zone over time. This calls for careful management, such as periodic flushing or use of high-quality water and balanced fertilization to avoid salinity buildup. Further research is warranted to investigate soil salinity dynamics in lined beds and whether any amendments (e.g., gypsum or organic matter) are needed to maintain soil health.

For researchers, the study opens several avenues. One is the combination of the lined trench-bed with other water-saving practices: for instance, could mulching be applied within a lined bed to further enhance WUE? Also, exploring deficit irrigation regimes finer than 50% ETc (e.g., 40% or 30% ETc) in lined beds could identify the threshold beyond which yields sharply drop. The study was conducted inside a shade-net structure; repeating similar trials in open-field conditions or different climates would help generalize the findings.

## Conclusion

The lined trench-bed system significantly enhanced cucumber drought tolerance and water productivity under semi-arid, protected conditions. It allowed plants to maintain growth, nutrient status, and yield at 50% ETc irrigation by conserving every drop of water in the root zone. These results not only confirm the initial hypotheses but also contribute novel insights into root-zone management as a drought mitigation strategy. Compared to conventional beds, the lined trenches produced a crop that was physiologically less stressed (higher LRWC, chlorophyll, lower MDA) and agronomically more productive per unit water (higher WUE). For practitioners, adopting such a system could mean achieving satisfactory yields with half the water, a compelling proposition in regions facing water scarcity. For researchers, the study highlights the importance of looking below-ground at soil–water dynamics and encourages further exploration of bed design, irrigation scheduling, and crop responses in dryland horticulture. Ultimately, innovations like the lined trench-bed bring us a step closer to sustainable, water-efficient crop production in the challenging environments of arid and semi-arid zones.

## Data Availability

The data presented in this study are available upon request from the corresponding author.

## References

[1] Iqbal, R. et al. Potential agricultural and environmental benefits of mulches—a review. *Bull. Natl. Res. Centre*. **44** (1), 44–41 (2020). (2020).

[2] Kafle, A. et al. Influence of deficit irrigation and Biochar amendment on growth, physiology, and yield of cucumber in West Texas. *Sci. Rep.* **15**, 1–17 (2025). 40113936 10.1038/s41598-025-94113-y PMC11926378

[3] Parkash, V. et al. Effect of deficit irrigation on root growth, soil water depletion, and water use efficiency of cucumber. *HortScience* **56**, 1278–1286 (2021).

[4] Guo, Y. et al. Deficit irrigation of greenhouse cucumber reduces mineral leaching and improves water use efficiency while maintaining fruit yield. *Nitrogen 2025*. **6, Page 18** (6), 18 (2025).

[5] El-Beltagi, H. S. et al. Mulching as a sustainable water and soil saving practice in agriculture: A review. *Agron. 2022*. **12**, 1881 (2022).

[6] Kapoor, R. et al. Water and nutrient economy in vegetable crops through drip fertigation and mulching techniques: a review. *J. Plant. Nutr.* **45**, 2389–2403 (2022).

[7] Kaya, C., Higgs, D. & Kirnak, H. Influence of polyethylene Mulch, irrigation Regime, and potassium rates on field cucumber yield and related traits. *J. Plant. Nutr.* **28**, 1739–1753 (2005).

[8] Kirnak, H. & Demirtas, M. N. Effects of different irrigation regimes and mulches on yield and macronutrition levels of drip-irrigated cucumber under open field conditions. *J. Plant. Nutr.* **29**, 1675–1690 (2006).

[9] Chen, B., Liu, E., Mei, X., Yan, C. & Garré, S. Modelling soil water dynamic in rain-fed spring maize field with plastic mulching. *Agric. Water Manag*. **198**, 19–27 (2018).

[10] Vijay, V. et al. Review of Large-Scale Biochar Field-Trials for soil amendment and the observed influences on crop yield variations. *Front. Energy Res.* **9**, 710766 (2021).

[11] Piccoli, I. et al. Hydrogels for agronomical application: from soil characteristics to crop growth: a review. *Agron. Sustainable Dev. 2024*. **44:2 44**, 1–23 (2024).

[12] Lahbouki, S., Meddich, A., Ben-Laouane, R., Outzourhit, A. & Pari, L. Subsurface water retention technology promotes drought stress tolerance in Field-Grown tomato. *Energies 2022*. **15**, 6807 (2022).

[13] Pari, L., Stefanoni, W., Palmieri, N. & Latterini, F. Assessing the performance of a subsurface water retention system (SWRS) prototype: first evaluation of work productivity and costs. *Inventions 2022*. **7**, 25 (2022).

[14] Jackson, M. L. & Jackson, M. L. Soil Chemical Analysis. Prentice-Hall of India Pvt. Ltd., New Delhi, 498. - References - Scientific Research Publishing. (1967). https://www.scirp.org/reference/referencespapers?referenceid=2394508 (1976).

[15] BLACK, TA, GARDNER & WR & THURTELL GW. The prediction of Evaporation, Drainage, and soil water storage for a bare soil. *Soil Sci. Soc. Am. J.* **33**, 655–660 (1969).

[16] Abdou, M. A. A. et al. Using deficit irrigation strategies and adding sugarcane waste Biochar as a sustainable material to sandy soils for improving yield and water productivity of cucumber. *Sustain. 2024*. **16, Page 4856** (16), 4856 (2024).

[17] Allen, R. G., Pereira, L. S. & Raes, D. Crop Evapotranspiration-Guidelines for Computing Crop Water Requirements-FAO Irrigation and Drainage. *Irrigation and Drainage Paper 56. FAO, Rome* 300, (1998).

[18] Kumar, P., Khapte, P. S., Singh, A. & Saxena, A. Optimization of Low-Tech protected structure and irrigation regime for cucumber production under hot arid regions of India. *Plants 2024*. **13**, 146 (2024). 10.3390/plants13010146 PMC10780501 38202454

[19] Van der Lugt, G., Holwerda, H. T., Hora, K. & Bugter, M. *Nutrient Solutions for Greenhouse Crops* (Eurofins Agro, Geerten van der Lugt, 2020).

[20] Schrader, J. et al. Leaf size Estimation based on leaf length, width and shape. *Ann. Bot.* **128**, 395–406 (2021). 34157097 10.1093/aob/mcab078 PMC8414912

[21] Bremner and Mulvaney. Bremner, *J.M. and* Mulvaney, C.S. (*) Nitrogen-Total. In Methods of Soil Analysis. Part 2. Chemical and Microbiological Properties, Page, A.L., Miller, R.H. and Keeney, D.R. Eds., American Society of Agronomy, Soil Science Society of America, Madison, Wisconsin, 595–624. - References - Scientific Research Publishing*. (1982). https://www.scirp.org/reference/ReferencesPapers?ReferenceID=181829 (1982).

[22] Knudsen, D., Knudsen, P. P., Peterson, D. & Pratt, P. P. G. A. and, G.A. and Lithium, Sodium and Potassium. In Page, A.L., Ed., Methods of Soil Analysis, American Society of Agronomy, Madison, 225–246. - References - Scientific Research Publishing. (1982). https://www.scirp.org/reference/ReferencesPapers?ReferenceID=2427154 (1982).

[23] Gavlak, R., Gavlak, K. A. J., Horneck, R., Miller, D. & Kotuby-Amacher, J. H. D.M. R. O. and,R.O. and Soil, Plant and Water Reference Methods for the Western Region. WCC-103 Publication, Fort Collins. - References - Scientific Research Publishing. (2003). https://www.scirp.org/reference/referencespapers?referenceid=1570658 (2003).

[24] WEATHERLEY, P. E., STUDIES IN THE WATER & RELATIONS OF THE COTTON PLANT. I. THE FIELD MEASUREMENT OF WATER DEFICITS IN LEAVES. *New Phytol.* **49**, 81–97 (1950).

[25] Bates, L. S., Waldren, R. P. & Teare, I. D. Rapid determination of free proline for water-stress studies. *Plant. Soil.* **39**, 205–207 (1973).

[26] Heath, R. L. & Packer, L. Photoperoxidation in isolated chloroplasts: I. Kinetics and stoichiometry of fatty acid peroxidation. *Arch. Biochem. Biophys.* **125**, 189–198 (1968). 5655425 10.1016/0003-9861(68)90654-1

[27] Shehata, S. A. et al. Grafting enhances drought tolerance by regulating stress-responsive gene expression and antioxidant enzyme activities in cucumbers. *BMC Plant. Biol.* **22**, 1–17 (2022). 35987604 10.1186/s12870-022-03791-7 PMC9392319

[28] Barzegar, T., Ghoreyshi, S. M., Nekounam, F., Nikbakht, J. & Ghashghaie, J. Growth, Yield, and physiological responses of cucumber (Kish F1 Hybrid) grafted onto African horned cucumber (Cucumis metuliferus L.) rootstock under deficit irrigation stress. *Russ. J. Plant Physiol.* **71**, 1–10 (2024).

[29] Farooq, M., Wahid, A., Kobayashi, N., Fujita, D. & Basra, S. M. A. Plant drought stress: Effects, mechanisms and management. *Sustainable Agric.* **29**, 153–188 (2009).

[30] Cheraghi, M., Mousavi, S. M. & Zarebanadkouki, M. Functions of rhizosheath on facilitating the uptake of water and nutrients under drought stress: A review. *Plant and Soil* 491, 239–263 (2023). (2023).

[31] Wahab, A. et al. Plants’ Physio-Biochemical and Phyto-Hormonal responses to alleviate the adverse effects of drought stress: A comprehensive review. *Plants 2022*. **11, Page 1620** (11), 1620 (2022). 10.3390/plants11131620 PMC9269229 35807572

[32] Yaghi, T., Arslan, A., Naoum, F. & Cucumber Cucumis sativus, L.) water use efficiency (WUE) under plastic mulch and drip irrigation. *Agric. Water Manag*. **128**, 149–157 (2013).

[33] Abdelfattah, A. & Mostafa, H. Potential of soil conditioners to mitigate deficit irrigation impacts on agricultural crops: A review. *Water Resour. Manage*. **38**, 2961–2976 (2024).

[34] Haque, M. A., Jahiruddin, M. & Clarke, D. Effect of plastic mulch on crop yield and land degradation in South coastal saline soils of Bangladesh. *Int. Soil. Water Conserv. Res.* **6**, 317–324 (2018).

